# School Playground Surfacing and Arm Fractures in Children: A Cluster Randomized Trial Comparing Sand to Wood Chip Surfaces

**DOI:** 10.1371/journal.pmed.1000195

**Published:** 2009-12-15

**Authors:** Andrew W. Howard, Colin Macarthur, Linda Rothman, Andrew Willan, Alison K. Macpherson

**Affiliations:** 1 Division of Orthopaedic Surgery, Hospital for Sick Children, Toronto, Ontario, Canada; 2 Child Health Evaluative Sciences, Hospital for Sick Children, Toronto, Ontario, Canada; 3 Bloorview Kids Rehab, Toronto, Ontario, Canada; 4 Department of Kinesiology, York University, Toronto, Canada; University of Queensland, Australia

## Abstract

**Background:**

The risk of playground injuries, especially fractures, is prevalent in children, and can result in emergency room treatment and hospital admissions. Fall height and surface area are major determinants of playground fall injury risk. The primary objective was to determine if there was a difference in playground upper extremity fracture rates in school playgrounds with wood fibre surfacing versus granite sand surfacing. Secondary objectives were to determine if there were differences in overall playground injury rates or in head injury rates in school playgrounds with wood fibre surfacing compared to school playgrounds with granite sand surfacing.

**Methods and Findings:**

The cluster randomized trial comprised 37 elementary schools in the Toronto District School Board in Toronto, Canada with a total of 15,074 students. Each school received qualified funding for installation of new playground equipment and surfacing. The risk of arm fracture from playground falls onto granitic sand versus onto engineered wood fibre surfaces was compared, with an outcome measure of estimated arm fracture rate per 100,000 student-months. Schools were randomly assigned by computer generated list to receive either a granitic sand or an engineered wood fibre playground surface (Fibar), and were not blinded. Schools were visited to ascertain details of the playground and surface actually installed and to observe the exposure to play and to periodically monitor the depth of the surfacing material. Injury data, including details of circumstance and diagnosis, were collected at each school by a prospective surveillance system with confirmation of injury details through a validated telephone interview with parents and also through collection (with consent) of medical reports regarding treated injuries. All schools were recruited together at the beginning of the trial, which is now closed after 2.5 years of injury data collection. Compliant schools included 12 schools randomized to Fibar that installed Fibar and seven schools randomized to sand that installed sand. Noncompliant schools were added to the analysis to complete a cohort type analysis by treatment received (two schools that were randomized to Fibar but installed sand and seven schools that were randomized to sand but installed Fibar). Among compliant schools, an arm fracture rate of 1.9 (95% confidence interval [CI] 0.04–6.9) per 100,000 student-months was observed for falls into sand, compared with an arm fracture rate of 9.4 (95% CI 3.7–21.4) for falls onto Fibar surfaces (*p*≤0.04905). Among all schools, the arm fracture rate was 4.5 (95% CI 0.26–15.9) per 100,000 student-months for falls into sand compared with 12.9 (95% CI 5.1–30.1) for falls onto Fibar surfaces. No serious head injuries and no fatalities were observed in either group.

**Conclusions:**

Granitic sand playground surfaces reduce the risk of arm fractures from playground falls when compared with engineered wood fibre surfaces. Upgrading playground surfacing standards to reflect this information will prevent arm fractures.

**Trial Registration:**

Current Controlled Trials ISRCTN02647424

*Please see later in the article for the Editors' Summary*

## Abstract 2

In a randomized trial of elementary schools in Toronto, Andrew Howard and colleagues show that granitic sand playground surfaces reduce the risk of arm fractures from playground falls when compared with wood fiber surfaces.

## Editors' Summary

**Background:**

Playgrounds and outdoor play equipment provide children with a place to let steam off, play creatively, socialize, and learn new skills. And, in a world where childhood obesity is a burgeoning problem, playgrounds provide a place where children can be encouraged to exercise. But playgrounds are not without hazards. Even in well-maintained and well-run facilities, children can hurt themselves by falling off climbing frames, monkey bars, and other equipment or by falling from standing height during playground games such as tag. In the US alone, more than 200,000 children are treated in emergency departments for injuries sustained in playgrounds every year and about 6,400 children are admitted to hospitals because of playground injuries, most of which are bone fractures (broken bones). In fact, playground injuries in the US are more severe and have a higher hospital admission rate than any other sort of child injury except those involving vehicles.

**Why Was This Study Done?:**

Children who fall off playground equipment are nearly four times as likely to break a bone (often in an arm) as children who fall from standing height. To reduce the number of fractures that occur in playgrounds, some governments have limited the height of playground equipment. Some have also set standards for the type of surfaces installed in playgrounds and for the depth of sand or engineered wood fiber in loose fill surfaces. These standards are based on laboratory tests in which headforms (artificial heads) are dropped onto surfaces. However, these tests provide no information about the ability of different surfaces to prevent broken arms and other specific injuries in the real world. In this cluster randomized trial (a study in which groups of people are randomly assigned to receive different interventions), the researchers compare the rates of arm fractures in elementary (primary) school playgrounds in Toronto (Canada) that have wood fiber surfacing with the rates in playgrounds that have granite sand surfacing.

**What Did the Researchers Do and Find?:**

The researchers randomly assigned 37 elementary schools that had qualified for school board funding for replacement playground equipment to receive either wood fiber (19 schools) or granite sand surfacing (18 schools) in their playgrounds. 19 of the schools complied with their randomization (12 installed fiber and seven installed sand); two schools installed sand although they were randomized to install fiber and seven schools installed fiber instead of sand. The researchers evaluated the playgrounds and their surfaces several times during the 2.5-year study and collected data on how playground injuries happened and types of injuries from the schools, parents, and medical reports. Among the schools that complied with randomization, falls from height into sand resulted in 1.9 arm fractures per 100,000 student-months whereas falls into fiber resulted in 9.4 arm fractures per 100,000 student-months. Arm fracture rates and other injury rates were also higher for falls from height into fiber than into sand when all the schools that had installed new surfaces were considered. However, the rates of arm fracture and other injuries that did not involve a fall from height did not vary between surfaces.

**What Do These Findings Mean?:**

The accuracy of these findings is limited by the small number of arm fractures that occurred during the trial—only 20 children who fell into fiber and two who fell into sand broke an arm. The accuracy of the findings may also be limited by the failure of many schools to comply with randomization although the researchers found no obvious differences between the schools that did and did not comply with randomization that might have affected the trial's outcome. However, even with these limitations, the findings of this real-world study indicate that granitic sand surfaces substantially reduce the risk of arm fractures and other injuries caused by falls from playground equipment when compared with wood fiber surfaces. Thus, because falls from playground equipment are more likely to cause a fracture than falls from standing height, if playground surfacing standards are adjusted to reflect the findings of this study (that is, if sand surfaces are recommended in preference to wood fiber surfaces), many arm fractures in children should be prevented.

**Additional Information:**

Please access these Web sites via the online version of this summary at ttp://dx.doi.org/10.1371/journal.pmed.1000195.

- Safe Kids Canada provides information about playground safety and other aspects of childhood safety (in English and French)
- Safe Kids Worldwide is a global network of organizations whose mission is to prevent accidental childhood injury (in English and Spanish)
- The Nemours Foundation, a nonprofit organization for child health, provides information for parents on playground safety
- The Royal Society for the Prevention of Accidents provides information on the safety of indoor and outdoor play areas
- The US Centers for Disease Control and Prevention provides fact sheets on playground injuries
- The US Consumer Product Safety Commission also has information on playground safety, including resources designed for children such as The Further Adventures of Kidd Safety and Little Big Kids, a booklet on play safety written by children for children

## Introduction

Playground fractures—severe upper extremity fractures among 5–9 y olds resulting from a fall off a climbing frame, monkey bar, or similar equipment—are easily recognized by emergency department physicians and orthopaedic surgeons. The irony of injury risk in an environment specifically designed to promote healthy active play is clear.

In the US, a prospective cohort study found that playground injuries were more severe and had a higher admission rate than all other child injury mechanisms except transportation [1]. More than 213,700 children are treated in US emergency departments annually for playground equipment injuries. Of these injuries, 35% are fractures and 75% of the children were injured by falling. Three percent of playground injuries require admission to hospital, some 6,400 children per year, of whom 92% have fractures [2]. Equipment falls have a 3.9 times greater odds of severe fracture compared with playground fractures from standing height falls [3].

Major determinants of playground fall injury risk include fall height and surface area, with previous case-control studies reporting injury odds of 2.6–3 for falls from excessive heights, and injury odds of 2.3–18.2 for falls onto suboptimal surfaces [4]–[7]. Surfacing type and depth standards exist in Canada, the United States, Britain, Australia, and New Zealand [8]–[12]. These standards permit loose fill surfaces including sand and engineered wood fibre. Surface performance is based on laboratory assessments using dropped headforms, in which wood fibre tends to perform better than sand. Application of Canadian standards can identify schools with higher injury rates, and replacement of noncompliant playgrounds with those meeting standards reduces expected playground injuries by 50% [13]. Surfacing standards, however, are not based on epidemiological data or actual injury experience, nor do they incorporate test methods specific to arm fracture prevention [14],[15]. Since arm fractures from falls from heights are common and often serious, assessments of the real world performance of surfaces based on specific injury outcomes are required [2].

In 2003, there was a unique opportunity to design a real-world randomized trial of school playground surfaces in Toronto, Canada. The Toronto District School Board (TDSB) was planning to resurface 37 schools as part of a larger program to retrofit and replace unsafe school board playground equipment, and agreed to randomize the surfacing installed. TDSB uses both wood fibre and sand routinely, but does not use permanently installed rubber as it is too expensive for routine use. The primary objective was to determine if there was a difference in playground upper extremity fracture rates in schools with Fibar (wood fibre) surfacing compared to school playgrounds with granite sand surfacing. The secondary objectives were to determine if there were differences in overall playground injury rates or in head injury rates in school playgrounds with Fibar surfacing compared to school playgrounds with granite sand surfacing.

## Methods

### Participants

Schools were eligible for inclusion if they were due for replacement of playground equipment and surfacing in the 2003/2004 school year. Thirty-seven elementary schools (of 365 in total) in the TDSB met this criterion. The total student population attending these schools was 15,074 children. These schools were representative of all elementary schools in the TDSB on the basis of size and socioeconomic status. School principals gave consent for data collection at their school. Ethics approval was obtained from the Hospital for Sick Children.

### Intervention

The intervention was directed at the school level, with either Fibar or granite sand surfacing installed in the school playgrounds. Each school in the study replaced the playground equipment as well as the impact absorbing surface. All equipment and surfacing installed were compliant with Canadian Standards Association standards for playgrounds. Each of the surface materials were provided from a single supplier and were installed by TDSB staff according to manufacturer's instructions and Canadian Standard's Association guidelines. Each playground and surface was independently evaluated by a qualified third party playground inspector following installation and annually thereafter. These independent evaluations included full assessment of all structures and surfaces according to CSA guidelines; specifically headform drop testing was performed on all installed surfaces and all passed. Routine maintenance of each playground was carried out by school custodial staff on a daily or weekly basis, with monthly written reports on playground condition including surface condition submitted to the school board.

### Outcomes

The primary outcome was arm fracture rates per 100,000 student-months from falls onto the playground surfaces. Data were collected on arm fractures from other mechanisms, and on other types of playground injuries from other mechanisms. Specific attention was paid to collecting complete data about head injuries as a secondary outcome because of their importance, and because head injury criteria form the current basis for playground surfacing standards.

### Data Collection

#### Injury data

All injury data were collected at the school level via the Ontario School Board Insurance Exchange (OSBIE) incident reports. OSBIE reports are routinely completed by school staff, and OSBIE policy states that incident reports are to be filled out “when someone receives medical/dental attention.” An inservice with staff at each school was conducted by study staff to ensure that all fractures were reported. The presence of the study, the definition and symptoms and signs of a fracture, and the correct way of filling in an OSBIE form were reviewed with groups of teachers and staff at each school. Reminder posters and bone-shaped stress balls with contact information for the study were left at each school. Study staff visited each school physically on a quarterly basis and maintained an ongoing telephone or e-mail relationship with the school staff member responsible for injury reports. OSBIE reports were forwarded to the study coordinator by the school staff and were double checked against the central OSBIE database to ensure no forms were missing.

Parents of children who were injured were contacted by telephone (after initial contact by letter) for consent and to collect further information regarding the injury. The interview questions were based on the validated questionnaire used by the Canadian Hospital Injury Research and Prevention Program (CHIRPP), which includes information such as when and where the injury occurred, the type of injury, and the treatment for the injury [16]–[18]. Medical record verification of the injury was also performed.

The definition of an upper extremity fracture was parental report of a physician's diagnosis of “break,” “fracture,” or “dislocation” in children who were x-rayed and required a cast, splint, or sling. The definition of a head injury included children with a physician's diagnosis of head injury, skull fracture, concussion, or brain injury. All fracture or head injury parent reports and medical records were reviewed and coded by an orthopaedic trauma surgeon (AWH) who was blinded to the circumstances of the fall and to the surfacing.

#### Compliance

In addition to the annual playground inspection obtained by the school board and daily/weekly maintenance and monthly inspection reports completed by the schools, each school was visited three times annually by study staff from the hospital. The type and amount of play equipment and the type and dimensions of impact absorbing surfaces present were recorded. Depth and condition of surface was assessed at 20–50 points around the playground equipment on each visit in order to confirm that surface material was present in adequate depths at falling and exit points. Depth measurements were done without notifying the school principals or maintenance staff prior to the visit, to ensure representative measurements.

#### Exposure to play

Exposure to play was controlled by limiting the data collection to supervised hours of play during the school day. Each school has the same policy and provides supervision for playground use during the lunch break, two 15-min recesses, and for 20 min before and after school. Each school has the same policy regarding playground use and outdoor play during wet or inclement weather. Playgrounds are not used under frozen conditions. All playgrounds are in the same city so all schools experienced the same weather. Injuries or fractures occurring on these playgrounds after supervised school hours, or on weekends, were specifically excluded because neither the exposure nor outcome could be systematically ascertained as they could be during school hours. Use of the play equipment area was measured at each school in the spring of 2006, by taking three 1-min counts of the number of children playing on or entering each piece of equipment, or standing on the falling surface for that equipment. These direct observations were made to ensure that the popularity of playing on equipment did not differ systematically according to the surface (Fibar or sand), but the primary measure of exposure to play a priori was person time, measured as student-months. We chose spring days with pleasant weather (not raining) to avoid influence of weather on use.

#### Sample size

Based on retrospectively collected data from 1999–2001, a baseline arm fracture rate of 40 per 100,000 student-months was estimated. A clinically significant difference would be a halving of this rate to 20 per 100,000 student-months. Estimating 410 students per school provided 820 student years (y) of data over the 2-y study. Hayes' method of sample size estimation for cluster randomization was used [19]. Setting alpha = 0.05 and power at 80% and *k* (coefficient of variation between clusters) at 0.2, we estimated that 17 clusters per arm or 34 schools in total would be required.

#### Randomization

A computer-generated random number list was used to conduct a simple cluster randomization. The 37 participating schools were assigned to either Fibar or granite sand groups. 19 schools were to receive Fibar and 18 were to receive granite sand. All students within the cluster were included in data analysis.

#### Statistical methods

Crude total playground injury rates (per 100,000 student-months) and arm fracture rates were calculated. Rates were calculated for all injuries, injuries that occurred when falling onto study surfaces under the playground equipment, and other injuries that occurred on the playground equipment that did not involve falling onto the surface (referred to as “other play equipment injuries”). A Poisson model was used to estimate the rates and variances at each school, and a random effects model was used to estimate pooled rates for each arm of the trial and to compare the rates between arms.

### Trial Protocol

The full trial protocol (Text S1) and CONSORT statement checklist (Text S2) appear in appendices.

## Results

### School Flow

**Figure 1:**
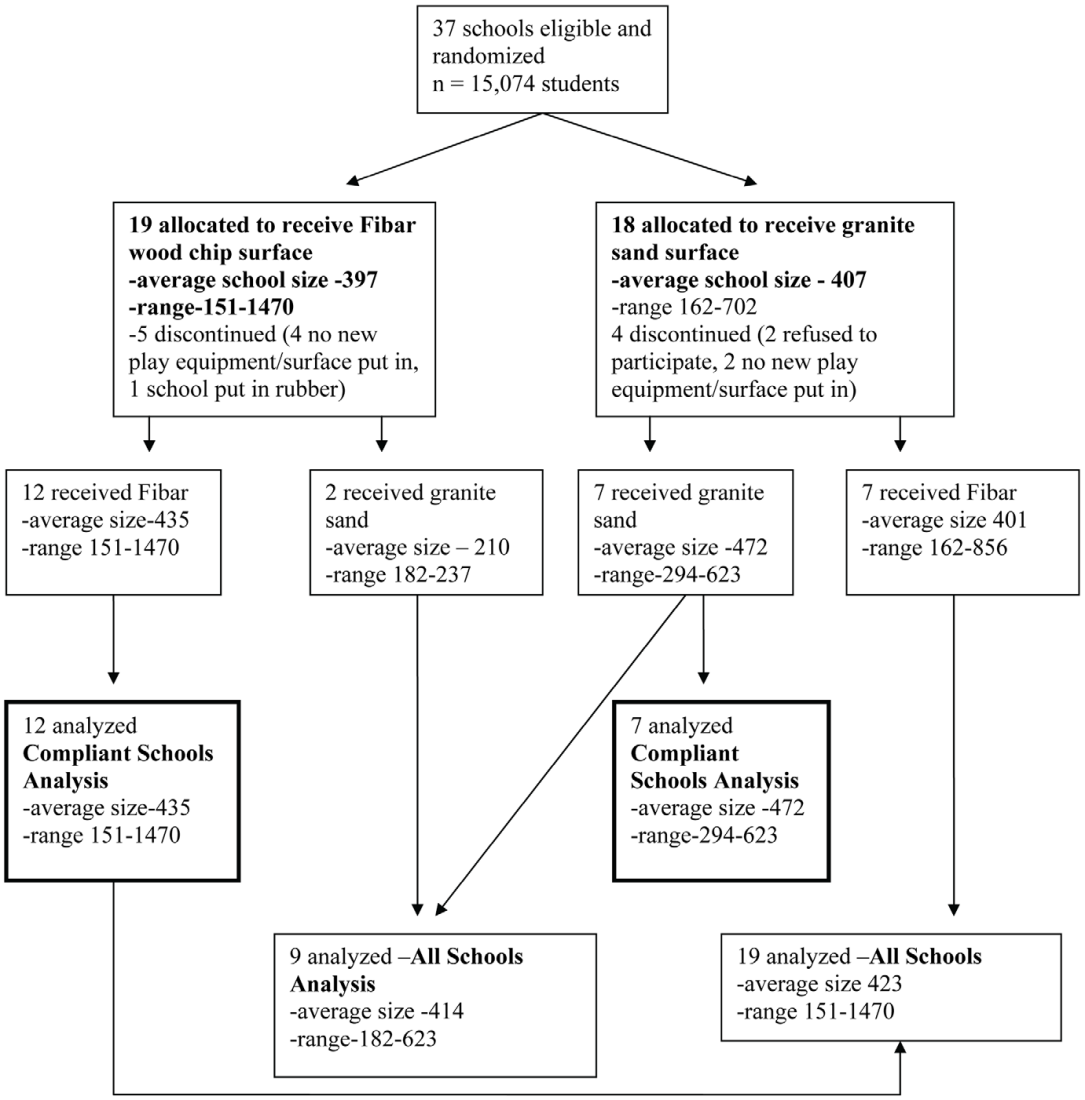
Flow diagram of a clustered randomized trial comparing injury rates in schools with Fibar (engineered wood fibre) and granite sand playground surfaces.

Figure 1 shows the flow of schools through the trial. Of 37 schools eligible for new play equipment, 19 were randomly allocated to receive a Fibar engineered wood fibre surface, and 18 were randomly allocated to receive a granitic sand surface. Each school principal received a proposal from the school board specifying the type of surfacing as well as the playground equipment to be replaced, and each school was allocated funding corresponding to the school board's proposal. A total of nine schools did not follow through (six did not install a new playground, one installed a playground with rubber matting, and two principals withdrew consent for participation that had been granted by a predecessor). The remaining 28 schools all installed new playground equipment and surfacing beginning in 2004, all installations were completed by January 2005. Data were collected at these 28 schools during the school months between January 2005 and June 2007.

Among 19 schools allocated to receive Fibar, 14 remained in the study sample of whom 12 installed Fibar and two installed sand. Among 18 schools allocated to receive sand, 14 remained in the study sample of whom seven installed sand and seven installed Fibar. Data were analyzed by intention to treat, by intervention among the compliant, and by intervention received at all schools. The intention to treat analysis was not judged clinically sensible because it attributed to the sand group a large number of fractures that were from falls onto Fibar. The analyses of the compliant schools (12 Fibar versus seven sand), and of all schools by intervention received (19 Fibar versus nine sand), are presented here. Schools that were compliant with the randomization did not differ in baseline characteristics from those that were not.

### Baseline Characteristics

Baseline information for each of the groups of schools is presented in Table 1. There were no differences between schools with sand surfaces and schools with Fibar surfaces in average school size or in socioeconomic status of school (expressed as learning opportunities index). The playground surface area per student, surface installation cost, and appropriate depth of surfacing did not differ between groups of schools. The number of pieces of specific types of equipment present, the maximum heights of equipment present, and the use of equipment also did not differ between groups of schools.

**Table 1:** Baseline information.

School Attribute	Compliant Schools (*n* = 19)		*p*-Value	All Schools (*n* = 28)		*p*-Value
	Sand (*n* = 7)	Fibar (*n* = 12)		Sand (*n* = 9)	Fibar (*n* = 19)	
**Total student-months**	81,002	129,870	—	91,126	196,684	—
**Learning opportunities index (mean)**	0.49	0.59	0.741	0.50	0.51	0.506
**Playground surface characteristics**						
**Total playground surface size (m^2^)**	3,341.5	5,157.8	—	3,870.5	6,863.3	—
**Playground surface per 100 student-months (m^2^)**	4.1 m^2^	4.0 m^2^	—	4.3 m^2^	3.5 m^2^	—
**TDSB surface installation costs (mean/school, CAD$)**	$7,721.29	$6,850.75	0.696	$7,494.33	$6,131.11	0.442
**Total number of depth measurements taken**	460	1,165	0.170	657	1,565	0.512
**Number depth measurements taken (%) with appropriate depth (≥17.78 cm)**	349 (75.9%)	845 (72.5%)	—	485 (73.8%)	1,176 (75.1%)	—
**Playground equipment characteristics**						
**Equipment to hang from**	17 (16.0%)	38 (14.6%)	0.72	22 (15.4%)	50 (15.1%)	0.94
**Slides**	19 (17.0%)	47 (18.0%)	0.99	26 (18.2%)	56 (16.9%)	0.74
**Vertical ladders and climbers**	42 (39.6%)	119 (45.6%)	0.30	60 (42.0%)	154 (46.5%)	0.4
**Horizontal steps**	11 (10.4%)	24 (8.1%)	0.73	15 (10.5%)	27 (8.2%)	0.41
**Misc** a	17 (16.0%)	33 (12.6%)	0.39	20 (14.0%)	44 (13.3%)	0.84
**Total pieces of equipment**	106	261	—	143	331	—
**Average height (m)**	2.98	3.11	0.53	2.96	3.00	0.85
**Highest equipment** b **(range)**	(2.13–3.56)	(1.88–3.79)	—	(2.13–3.68)	(1.88–3.79)	—
**Monkey bars >2.2 m (mean/school)**	0.43	0.17	0.33	0.33	0.16	0.45
**Use of equipment (students per minute)**	34	34	0.939	30	33	.962

a Includes swings, rockers, houses, cars, platforms, tic tac toes, top of tunnels, tables, stools, logs.

b Includes poles, tops of slides, railings of slides, top of monkey bars.

### Specific Injuries Recorded

A total of 259 injuries were reported for all schools, with 44 upper extremity fractures. 22 of the upper extremity fractures occurred falling onto the play equipment surface, and one occurred on play equipment but not as a result of falling from the equipment. Almost half of the upper extremity fractures were distal radius fractures, and 25% were distal humerus fractures. Among 20 upper extremity fractures from playground equipment falls into Fibar, the mean falling height was 213 cm. This height was not statistically different from the 218-cm mean falling height of the two upper extremity fractures that occurred from falls into sand (*p* = 0.77, *t*-test). Falling heights used here are the measured maximum height of the piece of equipment the child fell from. Among 22 upper extremity fractures occurring elsewhere on the playground, 19 were from standing height, and three were from falls off objects. One child sustained a concussion with a short loss of consciousness, but was not admitted to hospital. This injury did not occur in the playground equipment area and was the only head injury reported in either arm of the trial.

### Adjusted Injury Rates

Among schools compliant with the randomization, an arm fracture rate of 1.9 (95% confidence interval [CI] 0.04–6.9) per 100,000 student-months was observed for falls into sand, compared with an arm fracture rate of 9.4 (95% CI 3.7–21.4) for falls onto Fibar surfaces (*p*≤0.04905). Among all schools, the arm fracture rate was 4.5 (95% CI 0.26–15.9) per 100,000 student-months for falls into sand compared with 12.9 (95% CI 5.1–30.1) for falls onto Fibar surfaces. Rates are presented in Table 2. The total arm fracture rates were 8.7 (95% CI 3.4–17.4) per 100,000 students per month in schools with sand surfaces compared with 16.2 (95% CI 9.5–25.4) per 100,00 student-months for schools with Fibar surfaces. The risk of arm fractures because of falls not involving the playground equipment (about 7 per 100,000 student-months) was similar in the two groups (Fibar and sand), however, the risk of arm fracture from falls onto the playground surface was significantly lower in those schools with sand surfaces (1.9 fractures per 100,000 student-months) than in schools with wood fibre surfaces (9.4 fractures per 100,000 student-months).

**Table 2:** Adjusted estimates rate of injury per 100,000 student-months.

Injuries	Compliant Schools			All Schools		
	Sand	Fibar	*p*-Value	Sand	Fibar	*p*-Value
**All injuries**						
**Total**	127.2 (45.6–333.7)	101.3 (47.0–212.9)	0.81	144.1(59.0–334.1)	97.0 (53.7–172.6)	0.53
**Falling onto surface**	7.3 (1.4–22.6)	25.3 (11.3–54.7)	0.07	10.2 (2.5–30.0)	23.0 (11.2– 45.2)	0.17
**Other play equipment injuries (not falling)**	9.5(2.4–82.1)	9.0 (2.9–39.9)	0.95	14.0 (4.6–65.9)	10.7(4.8–27.4)	0.70
**Upper extremity fractures**						
**Total**	8.7 (3.4–17.4)	16.2 (9.5–25.4)	0.16	10.3 (3.3–25.1)	22.7 (12.3–41.3)	0.13
**Falling onto surface**	1.9* (0.04–6.9)	9.4 (3.7–21.4)	0.049	4.5 (0.26–15.9)	12.9 (5.1–30.1)	0.12
**Other play equipment injuries** a **(not falling)**	—	—	—	—	—	—

*: *p*≤0.05.

a ≤1 events, cannot calculate rates.

Considering all reported injuries, rather than just arm fractures, led to estimates of 7.3 (95% CI 1.4–22.6) injuries per 100,000 student-months from the mechanism of a fall into sand compared with 25.3 (95% CI 11.3–54.7) injuries per 100,000 student-months from the mechanism of falling onto Fibar. Injuries associated with the play equipment itself but occurring without falling off it were similar at 9.5 (95% CI 2.4–82.1) per 100,000 student-months at sand schools and 9.0 (95% CI 2.9–39.9) per 100,000 student-months at Fibar schools.

### Crude Injury Rates

Crude injury rates for each school are reported in Table 3. A slightly higher number of injuries was reported among schools with sand surfaces (106.2 per 100,000 student-months) than among schools with Fibar surfaces (92.4 per 100,000 student-months), whereas the rate of upper extremity fractures was markedly lower among schools with sand surfaces (1.2 versus 8.5 per 100,000 student-months).

**Table 3:** Crude (not adjusted for clustering) rates per 100,000 student-months.

All Injuries	Compliant Schools		All Schools	
	Sand	Fibar	Sand	Fibar
**Total**	106.2	92.4	111.8	79.8
**Falling onto surface**	4.9	23.1	6.6	19.8
**Other play equipment injuries (not falling)**	8.6	12.3	10.2	13.6
**Upper extremity fractures**				
**Total**	8.6	16.2	8.8	18.3
**Falling onto surface**	1.2	8.5	2.2	9.7
**Other play equipment injuries (not falling)**	0	0.8	0	0.7

Table 4 presents the characteristics of all injuries and arm fractures for the compliant schools and all schools together. The highest proportion of treatment locations for injuries on sand was at school followed by hospital emergency department, and for Fibar was hospital emergency department followed by school. Arm fractures were treated in the hospital emergency department. Among schools with sand surfaces 10.5% of all injuries reported were from a fall into sand, whereas among schools with Fibar surfaces 27.5% of injuries reported were from falls into Fibar (*p*<0.001, chi square). Among schools with sand surfaces 14% of arm fractures were from falls into sand, whereas among schools with Fibar surfaces 52% of arm fractures were from falls into Fibar.

**Table 4:** General characteristics of total injuries and arm fractures by received surface type.

Injuries	Compliant Schools (*n* = 206)			All Schools (*n* = 259)		
	Sand	Fibar	*p*-Value	Sand	Fibar	*p*-Value
**Total Injuries**	(*n* = 86)	(*n* = 120)	—	(*n* = 102)	(*n* = 157)	—
**Age (y) (mean, SD)**	9.69	8.03	0.002**	9.6 (2.28)	8.2 (1.8)	0.004**
**Male (%)**	64 (74.4%)	73 (61.3%)	0.050*	75(73.5%)	93 (59.6%)	0.022*
**Most severe injuries**						
**Superficial**	48 (60%)	50 (43.9%)	0.066	57 (60%)	58 (39.2%)	0.004**
**Fractures**	11 (13.8%)	25 (21.9%)	—	13 (13.7%)	42 (28.4%)	—
**Open wound/laceration**	8 (10.0%)	12 (10.5%)	—	10 (10.5%)	20 (13.5%)	—
**Most severe body part involved**						
**Face**	25 (29.1%)	23 (19.2%)	0.395	30 (29.4%)	25 (15.9%)	0.075
**Upper extremity**	21 (24.4%)	38 (31.7%)	—	24 (23.5%)	56 (35.7%)	—
**Scalp, skull, head**	13 (15.1%)	16 (13.3%)	—	16 (15.7%)	24 (15.3%)	—
**Lower extremity**	14 (16.3%)	13(10.8%)	—	16 (15.7%)	20 (12.7%)	—
**Treatment received**						
**School**	24 (41.4%)	25 (23.6%)	0.069	27 (38%)	30 (21.7%)	0.149
**Home**	2 (3.4%)	11 (10.4%)	—	5 (7%)	11 (8.0%)	—
**Doctor's office**	12 (20.7%)	29 (27.4%)	—	14 (19.7%)	32 (23.2%)	—
**Hospital emergency**	20 (34.5%)	41 (38.7%)	—	25 (35.2%)	64 (46.4%)	—
**Admitted to hospital**	0	0	—	0	1 (0.5%)	—
**Injury result of falling onto play equipment surface**	9 (10.5%)	33 (27.5%).	0.000***	12 (11.8%)	43 (27.4%)	0.000***
**Upper extremity fractures**	(*n* = 7)	(*n* = 21)	—	(*n* = 8)	(*n* = 36)	—
**Age (mean SD)**	9.42	7.52	0.520	9.3 (1.4)	7.9 (1.9)	0.067
**Male (%)**	5 (71.4%)	13 (61.9%)	0.207	5 (62.5%)	21 (58.3%)	0.047*
**Treatment received**			0.346	—	—	—
**School**	0	0	—	0	—	0.792
**Home**	0	0	—	0	—	—
**Doctor's office**	0	1 (4.8%)	—	0	1 (2.8%)	—
**Hospital emergency**	7(100%)	20 (95.2%)	—	8 (100%)	34 (94.4%)	—
**Admitted to hospital**	0		—	0	1 (2.8%)	—
**Injury result of falling onto play equipment surface**	1 (14.3%)	11 (52.4%)	0.141	2 (25%)	20 (55.6%)	0.227

SD, standard deviation.

*: *p*≤0.05.

**: *p*≤0.01.

***: *p* = 0.001.

## Discussion

This study provides empirical evidence that granitic sand playground surfaces are better than engineered wood fibre playground surfaces at preventing upper extremity fractures from equipment falls. The risk of an arm fracture from a fall off playground equipment on to the surface was 4.9 times higher in schools with Fibar (9.4 per 100,000 student-months, 95% CI 3.7–21.4) compared with schools with granitic sand playground surfaces (1.9 per 100,000 student-months, 95% CI 0.04–6.9). This increased risk of arm fracture was also observed when schools were included in an analysis by intervention received. The analysis by intervention received should be interpreted as a prospective cohort study, but not as a randomized study. Risk of other injuries from falling onto the playground surface was also higher among schools with Fibar surfaces. The risk of arm fractures and injuries from mechanisms not involving a fall onto the surface was equal for schools with Fibar or sand surfaces.

The observed differences are more likely a result of the surfacing than of other factors. First, the study was randomized. Second, the schools in the two arms of the study were similar at baseline in all important variables including population, socioeconomic status, playground characteristics, playground maintenance, and exposure to play. Third, specific details of both the mechanism and the injury were carefully considered. We were able to distinguish arm fractures from playing outdoors away from the playground equipment from arm fractures occurring as a result of a fall from equipment onto the impact-absorbing surface. Arm fractures from free play away from the playground occurred at a similar rate of 7 per 100,000 student-months irrespective of the playground surface, whereas those from equipment falls onto the surface were significantly more common in schools with Fibar surfaces. Finally, data on all other injuries were collected to establish that there was no difference in injury reporting between groups of schools; in fact the schools with sand had a slightly higher overall reporting activity so it is unlikely any fractures were missed.

These differences are likely important. Our previous analysis of 1,070 fractures from playground falls treated in a single hospital showed that falls from playground equipment resulted in severe fractures (requiring manipulative or operative reduction) 3.9 times more often than did falls from a standing height on the playground [3].

Two limitations of this study are notable. One is that the overall rate of arm fractures was substantially lower in both arms of the study than we had anticipated from previous work within the same school board. This resulted in borderline levels of statistical significance despite the finding of point estimates of effect size being much higher than we had planned for. We ascribe this to two factors. First, prospective data collection with medical records verification has eliminated misclassification compared with previous retrospective data recorded by teachers at the time of the incident. In other words, presumed fractures may have been included in the fracture group previously, but were not in this study. Second, all of the playground equipment in the present study was new, complied with current standards, and was in a study of surfacing, so particular attention was paid both to installation and maintenance of the surfacing by the schools, the school board, and the study staff (as detailed in the methods section). We interpret this as further evidence that standards work, and surfacing works. Our depth measurements indicated a fairly high rate (70%–75%) of ongoing maintenance of the study surfacing throughout the study period, which is in marked distinction to previous injury-based reports, which have consistently found poorly maintained surfacing [4]–[7]. Our depth measurements and attenuation tests were not done following an injury, so are not directly comparable to studies using that design. Sherker et al. found that over 85% of Victoria playgrounds complied with recommended maximum equipment height and surface impact attenuation characteristics, but that only 4.7% complied with recommended surface depth [14]. A marked deterioration in surface depth and impact attenuation was seen after only 8 wk in wood-based loose fill surfaces [20]. The fact that both arms of our study had well maintained surfaces and lower injury rates than predicted could be interpreted as the importance of applying a standard and maintaining the surface. Both surfaces functioned better than we expected, but we interpret the data as showing that sand was safer.

The second limitation is that of 28 schools that installed a new playground and a new surface and complied with follow-up, only 19 installed the surface that was assigned randomly whereas nine crossed over to the other surface. This situation appeared asymmetrical, with seven schools assigned to sand installing Fibar, and only two schools assigned to Fibar actually installing sand. It is important to understand the decision making in Toronto schools to understand this phenomenon. Randomization was performed at the level of the school board who provided to individual schools both the proposal for a new playground and surfacing, and the actual funding. The funding can be used by the school for any locally determined variation on the proposal. Therefore, the parents' council, teacher input, and custodial staff input are taken into account before the school principal finally signs off on the implementation. While the school principals all gave consent to being in a randomized trial prior to randomization, it is clear that compliance with randomization did not override other local concerns. Some schools, for instance, installed outdoor gardens instead of playgrounds. Reasons given for installing Fibar instead of sand were not about safety but included considerations of sand being tracked into schools, sand being thrown by children, and sand being soiled by cats and dogs. We recorded no injuries from thrown sand. Our data collectors, once sensitized to the issue of the potential for soiled sand, did not actually come across any incidents either by report or during inspection of the playground.

Our findings are consistent with prior case-control studies that provided evidence that compliant impact-absorbing surfaces reduced the risk of severe playground injury [4]–[7]. We have extended these findings substantially by a prospective experimental design, and by the demonstration that sand provides better protection against arm fractures than does wood fibre. This finding is consistent with an earlier study showing that fall injury rates were lower in schools with sand playgrounds than those with grass or asphalt [21]. It is also consistent with a well-designed case-control study showing that the arm fracture rates on playgrounds with bark surfaces were no different from those with concrete surfaces [22].

There was only one significant head injury that occurred in this study, which did not require any hospitalization. This number is consistent with other reports of a very low head injury risk but a substantial risk of arm fractures on modern playgrounds [23],[24]. Half of the arm fractures that we observed occurred from falls off play equipment onto the surfacing with the other half occurring as a result of standing height falls elsewhere on the playground. This is consistent with reports of school playground injuries from other countries [25]. If our data are representative, then the 160,000 annual emergency department visits in the US from playground falls might be reduced by 90,000 to 110,000 and the 5,900 playground fall fracture-related hospitalizations might be reduced by 3,900 to 4,700. (These estimates are based on population attributable risk calculations assuming that the risk ratios for sand and Fibar apply to the risk ratio between sand and the “average’ installed playground surface in the US).

We suspect that the fracture rates are lower on sand because of lower surface friction. Granitic sand as specified for this study has very uniform and very round particles, which maximize its fluid-like properties and minimize surface friction. During a fall the bone breaks because of tensile overload of the convex apex of the bend. A lower friction surface allows the hand to slide or sink limiting the bending moment and preventing a fracture. Playground surface friction has been shown substantially lower for sand than for Fibar playgrounds [26] and this likely explains the protective effect.

Playground fractures are a serious health problem created by an environment built specifically for children. Prior investigations have consistently shown height and surfacing to be important risk factors, but no study has prospectively investigated the effects of an intervention using injury outcomes. This investigation shows that the risk of an arm fracture was 4.9 times higher over an engineered wood fibre playground surface compared with a sand playground surface. Updating playground safety standards to reflect this information will reduce the most common and severe injuries seen on modern playgrounds, without limiting children's access to healthy outdoor play.

## Supporting Information

Text S1 **Full trial protocol.**
(0.10 MB DOC)

Text S2 **CONSORT statement checklist.**
(0.07 MB PDF)

## Abbreviations

CI: confidence interval
OSBIE: Ontario School Board Insurance Exchange
TDSB: Toronto District School Board
