# Supplementary material for: School Playground Surfacing and Arm Fractures in Children: A Cluster Randomized Trial Comparing Sand to Wood Chip Surfaces
Source: PLoS Med. 2009 Dec 15;6(12):e1000195. doi: 10.1371/journal.pmed.1000195 (PMC2784292; doi:10.1371/journal.pmed.1000195)
Supplement: Text S1 — Full trial protocol. (0.10 MB DOC) [file pmed.1000195.s001.doc]

# THE NEED FOR A TRIAL

## What is the problem to be addressed?

We propose a cluster randomized trial to determine which surface (engineered wood fibre or sand) better protects children from upper extremity fractures from school playground falls.

Fully 60 percent of Canadian children’s Emergency Department (ED) injury visits result from sports and leisure activities(1). Playground equipment injuries alone comprise ten percent of ED injury visits among 5 to 9 year old Canadian children (2). At Toronto’s Hospital for Sick Children from 1997 to 2002, 739 upper extremity fractures were seen from playground equipment falls, of which 331 (45%) required operative reduction. By comparison, 353 upper extremity fractures were seen from standing height playground falls, of which 49 (14%) required operative reduction (data source: Canadian Hospitals Injury Reporting and Prevention Program, CHIRPP, unpublished). In the US (3), a prospective cohort study found that playground injuries had a higher severity than any other child injury mechanism except transportation. This study also found that the admission rate (8%) for playground injuries was higher than that for any other child injury mechanism except transportation. Phelan (4) reported 920,000 emergency department visits in the US from 1992 to 1997 resulting from playground equipment falls, and concluded that “interventions targeting schools and 5 to 9 year old children may have the greatest impact on reducing emergency visits for playground injuries.” Chalmers (5), in a study in New Zealand, demonstrated that optimizing falling height and surface conditions would decrease playground injury ED visits by 45%.

The major determinants of playground injury reported consistently in case control studies are fall heights greater than 1.5 metres and poor surfacing. Falls of 1.5 metres and up had 2.0 to 4.1 times higher odds of significant injury than lower falls (5,6). Lack of impact absorbing surfaces increased injury odds 2.3 times in a New Zealand study (5). In Kingston Ontario, injury odds were 18 times higher for a fall onto a surface which did not meet CSA impact absorption standards (7).

Current CSA standards for playground surfaces can be met using permanently installed ground rubber, wood chips/wood fibers, or sand. Permanently installed rubber is too expensive for routine use in Toronto schools, but wood fiber and sand are both used routinely. Mechanical testing with dropped headforms favours wood fibre, but epidemiological evidence favours sand. The surfaces have never been compared in a study using injury as an outcome. To date, there have been no systematic reviews nor randomized trials addressing playground surfacing.

Surfacing type and depth standards are based on laboratory assessment of the maximum fall height before a fatal (200G) acceleration of a dropped headform occurs. This height is 11 feet for wood chips and 8 feet for fine sand (8). Wood fibre also has better impact attenuation performance than sand when wet or frozen (9). Surfacing standards, however, are not based on any epidemiological evidence or actual injury experience (5,10,11).

Real world playground injuries rarely conform to the test scenario of a direct fall producing a fatal head injury. The most common severe playground injuries are upper extremity fractures, which occur when a child falls onto an outstretched arm. Lower friction surfaces which permit the arm to slide decrease the fracture risk (12). Sand is both significantly softer and lower friction than engineered wood fiber playground surfaces (13). Falls onto sand playgrounds had the lowest injury rates (7 per 10,000 student years), compared with higher friction surfaces such as gravel (15/10000), grass (12/10000), mats (16/10000), or asphalt (44/10000) (14). Sand surfaces were associated with lower severity playground injuries than other surfaces in a Montreal study (15) and in this study neither fall height nor impact attenuation alone influenced injury severity. Decreased injury risk on low friction surfaces is also well documented for football (16,17), indoor handball (18), and army obstacle course training (19). By contrast, wood (bark) surfaces under playground equipment (10,11) had as high, or higher a fracture rate than concrete. In this study of 30,000 children, fracture rates were adjusted for exposure to play on each surface type. The vast majority of the fractures were upper extremity fractures.

**Rationale:** Loose fill playground surfaces are popular because they are an inexpensive way of meeting impact attenuation standards. Physical and epidemiological studies suggests that fractures may occur more often on higher friction fills (wood fibre) and less often on lower friction fills (sand), but the surfaces have never been compared using injury rates as an outcome. Real world performance of the surface will also depend on its behaviour in wet, frozen, or snowy conditions and on how children play in that particular surface. The most meaningful test of effectiveness of a surface is injury rates during real world use. A unique opportunity exists to perform a randomized trial which will guide standards development and allow evidence based injury prevention. This is the first randomized trial of a playground injury prevention intervention, is methodologically unique, and will lead directly to improved standards and improved health.

## What are the principal research questions to be addressed?

### Primary research question:

Is there a difference in playground upper extremity fracture rates in schools with Fibar® (wood fibre) surfacing compared to schools with granite sand surfacing?

### 1.2.2. Secondary research questions:

Are there differences in overall playground injury rates and in head injury rates in school playgrounds with Fibar® (wood fibre) surfacing compared to school playgrounds with granite sand surfacing?

## Why is a trial needed now?

Despite the importance of playground injuries, to date, there are no randomized trials of injury countermeasures. Published intervention studies are pre post studies without injury outcomes. Therefore, they cannot be used to guide decision making. By examining injury rates by type of surface, it may be possible to dramatically reduce the numbers and severity of children’s playground injuries. Last, there is a unique opportunity to perform this study within an existing program of playground replacement carried out by the Toronto District School Board. This means that a high quality randomized trial can be conducted with high leverage and modest cost. In the summer of 2000, the Toronto District School Board (TDSB) removed playground equipment that did not comply with newly revised Canadian Standards Association (CSA) standards (20). Subsequently, there has been a phased replacement of equipment. In the spring of 2003, retrofitting and replacement of equipment began in 37 school playgrounds that had partial equipment removal. Both wood (Fibar®) and granite sand surfaces are routinely used in TDSB schools. The Toronto District School Board agreed to randomize surfacing for these 37 schools, allowing an ideal opportunity to determine the relationship between surface type and playground injury rates. The $1.7M cost of replacing the playgrounds according to the randomization schedule was covered by the TDSB. Playground injuries can be identified from a prospectively recorded population based database (Ontario School Board Insurance Exchange - OSBIE). The playgrounds have a service life of 10 to 20 years, so outcomes from this randomized cohort can be assessed once funding is secured.

## Relevant Systematic Reviews

None (Medline, Cochrane, bibliographies of retrieved studies)

## How will the results of this trial be used?

Evidence from the trial will be incorporated into the next revision (2008) of the Canadian Standards Association standard for playgrounds, ensuring translation into action across Canada. Dr. Howard has presented to the CSA subcommittee on sports and leisure injuries in the past. There are thousands of school and municipal playgrounds across Canada which have loose surfaces that require regular maintenance and replacement. Evidence regarding the effectiveness of different surfacing materials will allow determination of the safest physical environment for Canadian children

Results of the trial will be shared with the Toronto District School Board as pre-arranged, and offered to the City of Toronto Department of Parks and Recreation and the Ontario Ministry of Education. The Ontario School Board Insurance Exchange will forward results to all school boards in Ontario and to their counterparts in other provinces. We also anticipate that there will be media interest in the results of this study. The Hospital for Sick Children’s Public Affairs Department will assist the investigators in developing a press release and media strategy. Safe Kids Canada, the leading knowledge translation organization for children’s injury prevention, will convey the results to its over 1500 partner organizations in communities across Canada. In addition, results of the trial will be published in a leading international journal and presented at the World Conference on Injury Prevention and Control.

# THE PROPOSED TRIAL

## What is the proposed trial design?

A two arm, cluster randomized trial of the effect of playground surface on playground injury rates will be performed. 37 Schools have been randomized, 19 to receive a wood fibre (Fibar®) playground surface and 18 to receive a granite sand playground surface. Playground injuries will be recorded prospectively for two school years following the intervention.

## What are the planned trial interventions?

### Fibar®

Manufacturer’s guidelines report that Fibar® is a shredded, Engineered Wood Fibre® that is guaranteed to perform for 20 years. It does not decompose and is not chemically treated. Fibar® is certified by the International Play Equipment Manufacturers Association (IPEMA) in accordance with ASTM F1292-99 and CAN/CSA Z614-98 impact tests. Fibar® is a component of the Fibar System 300®. This consists of wood fibers compacted to a specified depth (usually 12”) over FibarFelt® (100% polyester, non-woven geotextile fabric) and FibarDrain® drainage system. FibarMats® (rubber mats) are placed under each swing, slide exit and sliding pole.

Specific installation and maintenance instructions ensure that the material is properly distributed, properly drained, and topped up to maintain correct impact attenuation. Fibar is currently the most popular surfacing for newly replaced Toronto school playgrounds. However, the concerns raised by Mott (10,11) about the fracture rates on bark chips (equal to that on concrete) may possibly apply to Fibar because the materials are physically similar.

### Granite Sand

Granite sand consists of sub-angular or sub-rounded grains made from naturally weathered granite rock. It is washed to be free of all organics and silt and clay contaminants. It has a percolation rate greater than 30 inches per hour, allowing for excellent drainage. Granite sand is compliant in accordance with ASTM F1292-99 and CAN/CSA Z614-98. Manufacturer’s guidelines report that granite sand does not compact, does not react with water to form a hard surface and does not require rototilling. When children run in the sand, they push it back into the high traffic hollows that tend to form under swings and the bottom of slides, reducing the required maintenance in these areas.

Installation requires attention to drainage and depth. Maintenance involves daily inspection and occasional cleaning.

## What is the allocation method?

A computer generated random number list was used to assign the 37 participating schools to Fibar® or granite sand groups. School level covariates including student population, socioeconomic status, and playground size are balanced between the groups as shown in table 1 below:

Table 1: Results of Randomization:

|  | **Fibar Schools**  **N = 19**  **Mean (S.D.)** | **Granitic Sand Schools**  **N = 18**  **Mean (S.D.)** | **P-Value** |
| --- | --- | --- | --- |
| Number of Students | 409.2 (312.1) | 416.3 (195.4) | 0.934 |
| Learning Opportunities Index (LOI) | 0.55 (0.27) | 0.49 (0.29) | 0.533 |
| Size of Playground (m^2)^ | 18826.6 (22657.1) | 17142.8(22589.3) | 0.835 |
| TDSB Budget ($) | 42914.6 (31086.8) | 48658.70 (27426.2) | 0.555 |
| Cost of re-surfacing ($) | 7073.68 (1442.55) | 5688.9 (1179.9) | 0.003 |

Note: The TDSB Learning Opportunities Index 2002-03 combines information from the following variables: average and median income of families with school-aged children; parental education; proportion of lone-parent families; recent immigration; housing type (apartment, single detached housing); student mobility. It is the standard socioeconomic status measure for TDSB schools.

## What are the proposed methods for protecting against other sources of bias?

### Observer Bias

Identification of injuries is via Ontario School Board Insurance Exchange (OSBIE) incident report forms which are filled out by teachers. All schools use the same forms and have the same written policy for when a report is required (‘when someone needs medical or dental attention’). Inservices will take place at each school to remind teachers and staff of the threshold and process of completing the reports. Follow up of incident reports by a research assistant will rely on parent report of the injury type and severity. We do not anticipate that the accuracy of parent report will differ by surface type. The parent interview will be based on the Canadian Hospital Injury Research and Prevention Program (CHIRPP) questionnaire. CHIRPP is a national ED based injury surveillance system. With participation by all 10 Canadian children’s hospitals, CHIRPP injury data (provided by parents and physicians) have been shown to be reliable and valid (21-23). Finally, the primary outcome is upper extremity fractures, which we believe will be consistently reported across schools due to their severity.

### Co-interventions

The playground resurfacing program is part of a larger program that will also bring playground equipment up to CSA standards. Therefore, there may be modification of play structures within playgrounds. Such modifications, however, will be similar and randomizing the intervention will balance such cointerventions. We know that the total cost of replaced equipment is balanced between groups (table 1 in sec. 2.3). Actual equipment present at each playground will be surveyed, photographed, and measured (see appendix). Installation and maintenance of both surfaces will be done by the same TDSB employees working to CSA standards. Maintenance may differ systematically between groups if one surface is more costly or difficult to maintain, so actual surfacing depths will be measured during the followup period and treated as covariates.

### Contamination

Noncompliance with the assigned surfacing could occur if schools install a different surface to the one assigned, or install a different surface altogether (eg a fixed rubber surface). Analysis will be by intention to treat amongst randomized schools. Actual surface installed will be recorded for each school by direct observation during a site visit. Because both surfaces are approved, and the project has support from the TDSB, we anticipate that most schools will comply with the results of randomization. In addition, neighbourhood children may use school playground equipment outside school hours. Such use will not affect the results of the trial since data collection is based on incident reports completed during the time school is in session.

### Covariates

School population, playground size, socioeconomic status, and number of hours of play may influence injury rates although such effects would be independent of surfacing so it is simply necessary to balance the groups. The randomization has already been performed and school population, playground size, and socioeconomic status are balanced as would be expected with 37 clusters (see table 1 in sec 2.3). At each school, supervised outdoor play occurs for equal mandated time periods (totaling 2 hours) each day, weather permitting. Intensity of supervision will be addressed by calculating the student to teacher ratio at each school according to the playground supervision roster.

Weather will be the same for all schools, but it is possible that effect modification (different injury risks by surface in wet vs dry weather, or when frozen) could occur. Accordingly, rainfall and snowfall amounts will be recorded from Environment Canada web records and will be used in a secondary analysis as described below.

Depth of undersurface measurements will be done three times per year (September, January and June) of surfaces at all schools at falling points and slide exits. Falling heights will be recorded by surveying the equipment using diagrams and digital photos, and asking parents for the location of any injury fall. Depth measurements and falling heights will be used as covariates in the analyses (see appendix).

## What are the planned inclusion and exclusion criteria?

### Inclusion Criteria – Schools

Schools were eligible for inclusion if they were due for replacement of playground equipment and surfacing in the 2003/2004 school year. Thirty-seven elementary schools (of 365 in total) in the Toronto District School Board met this criterion. These schools are representative in terms of size and socioeconomic status. School principals will give consent for data collection at their school.

### Inclusion Criteria – Students

Any student injured on the playground (with sufficient severity to require an incident report) during school hours is eligible for inclusion. School hours when playgrounds are used include 15 minutes before the morning bell, 15 minutes each of morning and afternoon recess, an hour at lunch time, and 15 minutes after dismissal. During these times, supervision of play is provided at each school. The threshold for completing an OSBIE form is ‘when someone receives medical/dental attention’

### Exclusion Criteria – Students

Although we do not expect them, children with fractures through pathological bone (eg pre-existing tumor, osteoporosis) will be excluded.

## What is the proposed duration of the treatment period?

The TDSB planned the resurfacing of the playgrounds in the spring and summer of 2003, and the randomization was performed then. HSC ethics review board approval was obtained prior to randomization. School playground replacement began in the summer of 2003 with completion at all schools anticipated in the spring of 2004.

## What is the proposed frequency and duration of follow-up?

Data will be collected during two complete school years once funding is received. Data will be collected during supervised school hours (see inclusion criteria for definition).

## What are the proposed primary and secondary outcome measures?

### Primary Outcome Measure - Upper Extremity Fracture Rates

The primary outcome is an upper extremity fracture occurring on the school playground. The definition of an upper extremity fracture will be parental report of a physician’s diagnosis of ‘break’, ‘fracture’, or ‘dislocation’ in children who were x-rayed AND required a cast, splint or sling.

### Secondary Outcome Measure–Overall Playground Injury Rates

School playground injury is defined as any injury requiring completion of an incident report. OSBIE policy states that incident reports are to be filled out ‘when someone receives medical/dental attention’.

### Secondary Outcome Measure – Head Injury Rates

The definition of a head injury (based on parental report) will include children with a physician’s diagnosis of head injury, skull fracture, concussion, or brain injury.

## How will the outcome measures be measured at follow-up?

Ontario School Board Insurance Exchange (OSBIE) incident reports are routinely completed by teachers for any injury (playground or other) in every TDSB school. The threshold for completing an OSBIE form is ‘when someone receives medical/dental attention’. Inservices will be held for teachers at each school to remind them of the forms and the threshold for completion, as a means of standardizing the reporting.

The research assistant will receive new OSBIE incident reports describing playground injuries at each school on a weekly basis.

Parents of children who are injured will be contacted by telephone (after initial contact by letter) for consent and data collection. The CHIRPP form (see appendix) will be completed by telephone, such use has been validated (24). Additional telephone questions added to the CHIRPP form for details on the event and the injury are given in the appendix. The research assistant will work flexible hours allowing evening phone calls to parents where necessary. Hospital for Sick Children language line interpreter services will be used if a parent or caregiver has insufficient English.

To validate the injury data for severe injuries, health records (emergency sheet, and discharge sheet if admitted) will be obtained (with consent) for all children attending an emergency department or admitted to a hospital. ICD 10 diagnostic and procedure codes will be abstracted from emergency and inpatient records by trained coders.

We expect that the OSBIE incident reports will capture all severe injuries especially if there is an absence from school. A small number of injuries may have a delayed presentation (after school hours), and not be reported via the OSBIE incident reports. We expect that the school (ie the principals) would be notified if the injury was severe. Principals will be contacted monthly to ensure that no playground injuries have been missed

## What is the proposed sample size?

37 schools, each with approximately 410 students, will be followed for two years, yielding 30,000 student-years of observations. Sample size determinations for each outcome are provided below.

### Primary Outcome Measure

Based on OSBIE data from 1999-2001 we estimate a baseline arm fracture (lambda zero) rate of 4 per 1000 student years. A clinically significant difference would be a halving of this rate to 2 per 1000 student years (lambda one). This is consistent with reports of sand having ½ to 1/6 the injury rate of other surfaces (14). Each school of 410 students provides 820 student years (y) of data over the two year study. Hayes’ method of sample size estimation for cluster randomization was used (25), where the number of clusters (c) per arm is:

$$c=1+(z_{\alpha/2}+z_{\beta/2})^{2}[(\lambda_{0}+\lambda_{1})/y+k^{2}(\lambda_{0}^{2}+\lambda_{1}^{2})]/(\lambda_{0}-\lambda_{1})^{2}$$

Setting alpha = .05 and power at 80%,and k (coefficient of variation between clusters) at 0.2, we estimate that 17 clusters per arm or 34 schools in total will be required. 37 have been randomized so the study should have ample power. Ample power exists if the coefficient of variation is between 0.1 and 0.3 (see appendix).

### Secondary Outcome Measure

Based on OSBIE data from 1999-2002, we estimate a baseline injury rate of 15 reported injuries per 1000 student years. Anticipating a 33% reduction in injuries overall, and using the method above, we estimate that 15 clusters per arm or 30 schools in total will be required. 37 schools have been randomized so the study should have ample power, and would potentially be able to detect smaller differences.

Severe head injuries are rare events. They will be recorded because of their importance but we do not anticipate a statistically significant difference because of the small numbers.

## What is the planned recruitment rate?

All schools will be recruited at the beginning of the trial and we anticipate that all schools will agree to participate. Our previous work with OSBIE data suggests 15 playground injuries per 1000 students per year. Approximately 225 playground injuries per year will occur among 15,000 students, or 23 per month across the ten months school is in session. We believe that 90% of parents of injured children will agree to participate as there is only a minor inconvenience (one phone call) associated with participation and we have achieved this rate of participation in a previous telephone survey of play equipment injuries in Toronto (26).

## Are there likely to be any problems with compliance?

We do not anticipate problems with compliance because no ongoing actions are required by the schools after the playground surfaces are installed. Actual treatment installed will be recorded three times per year during site visits.

## What is the likely rate of loss to follow-up?

We expect minimal loss to follow up because parents of injured children are only required to complete one telephone interview. The research assistant will manage requesting and receiving medical records once consent is obtained.

## Give details of the planned analyses

### Primary outcome:

The primary analysis will include all randomized schools and upper extremity fractures as defined in Section 2.8.1. This effectiveness analysis will compare the rates of injuries in the two groups. A poisson model will be used since the outcomes are counts. For each school we will observe X, the number of injuries reported over the two-year period, and Y, the student-years accumulated over the two-year period. The estimated injury rate for each school is X/Y, with estimated variance X/Y^2^. Using methods proposed by Thompson et al. (27), an overall injury rate for each type of school (Fibar® or granite sand), along with the corresponding variances will be estimated. This method accounts for the extra variation due to the clustering. The injury rates can then be compared by a Z-statistic which is the difference in the two rates divided by the square root of the sum of the corresponding variances. Covariate adjustment for school level variables such as average age, proportion of males, school size, socioeconomic status, and surface depth will be facilitated using methods given by Raudenbush (28).

### Secondary outcome:

A similar analysis will be conducted for the secondary outcomes of all playground injuries and of head injuries. Fractures will be reanalyzed varying the severity threshold to consider only displaced fractures requiring reduction.

### Data Collection, Handling, Computerization and Analysis

Faxed copies of OSBIE incident reports will be sent from the school to the research assistant at the Hospital for Sick Children. The research assistant will then contact parents by letter to inform them of the study and then by telephone (evening where necessary) to obtain verbal consent to participate in the study. The CHIRPP form and appendix will be used to collect specific information regarding the injury. HSC telephone interpreter services will be used where necessary if English is not the first language. Faxed copies of emergency room or inpatient discharge sheets will be sent to the research assistant at HSC and will be coded using ICD 10 codes for diagnosis and procedures. A secure trial database will be maintained at The Hospital for Sick Children using ACCESS software. Completed data will be exported from ACCESS for analysis in SAS-PC.

## Are there any planned subgroup analyses?

Because the effect of the surface may be different in wet versus dry conditions, or in frozen versus thawed conditions, analysis by weather and by season will be undertaken. For analysis by weather, the data will be split into wet days (>= 2mm rainfall) and dry days and the analysis above will be repeated in each set. For analysis by season, the data will be split into winter (Dec 21 to March 21) and not winter (all other dates) and the analysis above will be repeated in each set.

## What is the proposed frequency of analyses?

The final analysis will be performed at the completion of the trial.

## Will the trial address any economic issues?

A cost-benefit analysis will be conducted from the Ontario provincial government perspective since both direct health care costs and education funding fall under the jurisdiction of the provincial government.

Direct health care costs will be calculated from OHIP perspective as follows: a) no visit – no cost. b) physician office visit only – GP minor assessment fee. c) emergency department visit – visit cost for ‘average’ Ontario ED visit according to ICD-10 procedure code assigned. d) inpatient admission – hospital cost for average Ontario hospital according to ICD-10 procedure code assigned. Data for the latter two categories will be obtained from the Ontario Case Costing Initiative ([www.occp.com](http://www.occp.com/)). If necessary, Hospital for Sick Children case costs per ICD-10 code will be used for uncommon diagnoses or procedures. Repeat hospitalizations and emergency department visits will be included. Followup clinic visits will be costed at a GP or specialist minor assessment fee as appropriate. Indirect and out-of-pocket costs will not be not included.

The cost of each surfacing will be based on itemized materials and labour contracts from playground suppliers plus labour costs paid by the Toronto District School Board and associated with installation. Maintenance costs will include annual costs of labour and supplies and will be provided yearly by the TDSB.

A decision analysis model will be used to compare the costs and outcomes of the two surfaces. Injury probabilities assigned to each surface will be based on injury rates observed in the trial. All costs will be expressed in 2004 Canadian dollars. A 10-year time horizon will be used. All costs beyond one year will be discounted at 3%. If one surface results in a better outcome at a lower cost, then the incremental cost savings in direct medical resource utilization per injury prevented will be reported. If one surface results in a better outcome at a higher cost, then the incremental cost per injury prevented will be calculated. Sensitivity analysis will be used to test the robustness of the result to variations in the underlying assumptions. The probability of health outcomes, cost of medical care for each health outcome, cost of intervention, and effectiveness of intervention will be varied over a clinically appropriate range using one-way sensitivity analysis.

The trial is being run from the Population Health Sciences program of the research institute at the Hospital for Sick Children. Accordingly, PhD trained health economists are available during data collection and analysis phases.

## What is the estimated cost and duration of the trial?

It is estimated that the data collection and analysis will cost a total of $100,734.40 over a two year period. The cost of replacing the playgrounds was $1,691,210, which is the school board’s in kind contribution. This contribution by the school board allows a high quality randomized trial to be conducted with high leverage and very modest cost. See attached budget for further details.

# Details of the Trial Team

## Trial Management

### Timelines

Random allocation of intended surfaces to schools occurred in the spring of 2003. HSC ethics review board approval for the study has already been obtained. School playground replacement began in the summer of 2003 and will be completed at all schools in the spring of 2004. The service life of each playground is estimated at 20 years. Data will be collected during two complete school years once funding is received. Data analysis will occur during the three months following completion of data collection.

### Investigators and their roles

Dr. Andrew Howard is a paediatric orthopaedic surgeon with an MSc in epidemiology. He holds CIHR principal investigator funding for two injury control projects. One is a pilot study to support a school based cluster randomized trial of child pedestrian injury prevention interventions in Kampala, Uganda. The other is an internet based health information project for surgeons and injury control workers in East Africa. He is coinvestigator on a large CIHR funded project to build injury research capacity in Canada. He has received peer reviewed grants from both CHIRPP and the Canadian Orthopaedic Foundation to study playground injuries. Dr. Howard has published widely on child occupant injury in motor vehicles. He has principal investigator grants from the Ontario Neurotrauma Foundation, Auto21 Network of Centres of Excellence, and the Ford motor company. Dr. Howard is a member of the National Expert Advisory Committee of Safekids Canada.

Dr. Colin Macarthur is an MD/PhD pediatric injury epidemiologist at the Hospital for Sick Children and is the director of the research institute at the Bloorview MacMillan centre. He has successfully run cluster randomized trials of bicycle education programs, and has published case – control studies defining playground injury risks. He has published validations and evaluations of the CHIRPP study (21-23). His methodological expertise and practical experience are directly relevant to the proposed trial. He is co-lead investigator on a recently funded CIHR grant for capacity enhancement in injury prevention research. Dr. Macarthur is also a member of the National Expert Advisory Committee of Safekids Canada.

Dr. Alison Macpherson is a PhD. researcher in child injury prevention. She has worked closely with Dr Howard and with the Toronto District School Board and the Ontario School Board Insurance Exchange on assessing the injury impact of a major playground equipment removal program which took place in 2000. A former recipient of the Emergency Health Services Fellowship, she has published several articles related to the effectiveness of bicycle helmet legislation on provincial head injury rates.

Dr. Andrew Willan is a senior PhD biostatistician with extensive experience in the design and analysis of randomized trials including cluster randomization designs.

Drs. Howard, Macarthur and Macpherson will be responsible for the design of the trial, and for hiring and overseeing the staff working out of the Hospital for Sick Children. Dr. Howard will oversee daily aspects of trial management. Dr. Willan will collaborate on the data analysis. All investigators will share in the interpretation and reporting of results. Dr. Howard will be responsible for dissemination of trial results, in particular to inform 2008 CSA standards for playgrounds.

### Trial Personnel

#### Research Coordinator (.2 FTE)

The coordinator will manage all aspects of implementation and staffing. She will design the study database and perform the data analysis. She will liaise with school and school board personnel during conduct of the trial. The research coordinator will also assist the dissemination of results in consultation with the principal investigators, including the preparation of manuscripts and scientific abstracts.

#### Research Assistant (.6 FTE)

The research assistant will be responsible for the day to day implementation of the study. This will include contacting the parents by mail and telephone and completing the CHIRPP forms, obtaining and coding health records, contacting the school principals, conducting the site inspections, and data cleaning and data entry.

# Ethical Issues

Hospital for Sick Children ethics review board approval has been obtained. Consent for data collection will be obtained from the schools with full disclosure of the trial design, purpose and outcomes. Schools will subsequently provide trial information to the parents at appropriate parent school meetings. Parents will be contacted first by letter, then by telephone within a week of a playground injury to request more detail regarding the injury and to complete the CHIRPP form. Consent will be obtained by telephone by the research assistant. Parents will be reassured that their participation is voluntary, and that the school and the TDSB will not be informed of their decision to participate. Prior contact by letter minimizes any coercive effect of ‘cold calling’ with a research request. We believe that in this situation, the research team is the best first contact because no member of the research team is providing direct patient care to any of the children in the study. Parents may not want the school to know if they agree to participate. Individual identity will be protected by using a study code in the database. Only the research coordinator will be able to link children to the database.

(1) Pickett W, Brison RJ, Mackenzie SG, Garner M, King MA, T L, et al. Youth injury data in the Canadian Hospitals Injury Reporting and Prevention Program: do they represent the Canadian experience? Inj Prev 2000;6305:9-15.

(2) Brown J. A comparison of injuries on various types of playground equipment. 1997:1-3.

(3) Yamamoto LG, Wiebe RA, Matthews WJ, Jr. A one-year prospective ED cohort of pediatric trauma. Pediatr Emerg Care 1991;7(5):267-74.

(4) Phelan KJ, K.J., Kalkwarf HJ,Lanphear BP. Trends and patterns of playground injuries in United States children and adolescents. Ambul Pediatr 2001;1(4373):227-33.

(5) Chalmers DJ, Marshall SW, Langley JD, Evans MJ, Brunton CR, Kelly AM, et al. Height and surfacing as risk factors for injury in falls from playground equipment: a case-control study. Inj Prev 1996;2(2):98-104.

(6) Macarthur C, Hu X, Wesson DE, Parkin PC. Risk factors for severe injuries associated with falls from playground equipment. Accident; Analysis and Prevention 2000 May;32(3):377-382.

(7) Mowat DL, Wang F, Pickett W, Brison RJ. A case-control study of risk factors for playground injuries among children in Kingston and area. Inj Prev 1998;4(1):39-43.

(8) Bond MT, Peck MG. The risk of childhood injury on Boston's playground equipment and surfaces. Am J Public Health 1993;83(5):731-3.

(9) Lewis LM, Naunheim R, Standeven J, Naunheim KS. Quantitation of impact attenuation of different playground surfaces under various environmental conditions using a tri-axial accelerometer. The Journal of trauma 1993 Dec;35(6):932-935.

(10) Mott A, Evans R, Rolfe K, Potter D, Kemp KW, Sibert JR. Patterns of injuries to children on public playgrounds. Arch Dis Child 1994;71(4):328-30.

(11) Mott A, Rolfe K, James R, Evans R, Kemp A, Dunstan F, et al. Safety of surfaces and equipment for children in playgrounds. Lancet 1997;349(9069):1874-6.

(12) Sosin DM, Keller P, Sacks JJ, Kresnow M, van Dyck PC. Surface-specific fall injury rates on Utah school playgrounds. American Journal of Public Health 1993 May;83(5):733-735.

(13) Chesney DA, Axelson PW. Preliminary test method for the determination of surface firmness. IEEE transactions on rehabilitation engineering : a publication of the IEEE Engineering in Medicine and Biology Society 1996 Sep;4(3):182-187.

(14) Sosin DM, Keller P, Sacks JJ, Kresnow M, van Dyck PC. Surface-specific fall injury rates on Utah school playgrounds. Am J Public Health 1993;83(5):733-5.

(15) Laforest S, Robitaille Y, Lesage D, Dorval D. Surface characteristics, equipment height, and the occurrence and severity of playground injuries. Injury prevention : journal of the International Society for Child and Adolescent Injury Prevention 2001 Mar;7(1):35-40.

(16) Orchard JW, Powell JW. Risk of knee and ankle sprains under various weather conditions in American football. Medicine and science in sports and exercise 2003 Jul;35(7):1118-1123.

(17) Powell JW, Schootman M. A multivariate risk analysis of selected playing surfaces in the National Football League: 1980 to 1989. An epidemiologic study of knee injuries. The American Journal of Sports Medicine 1992 Nov-Dec;20(6):686-694.

(18) Olsen OE, Myklebust G, Engebretsen L, Holme I, Bahr R. Relationship between floor type and risk of ACL injury in team handball. Scandinavian journal of medicine & science in sports 2003 Oct;13(5):299-304.

(19) Pope RP. Rubber matting on an obstacle course causes anterior cruciate ligament ruptures and its removal eliminates them. Military medicine 2002 Apr;167(4):355-358.

(20) Canadian Standards Association. A guideline on children's playspaces and equipment: A national standard of Canada. 1998.

(21) Macarthur C, Pless IB. Evaluation of the quality of an injury surveillance system. American Journal of Epidemiology 1999 Mar 15;149(6):586-592.

(22) Macarthur C, Pless IB. Sensitivity and representativeness of a childhood injury surveillance system. Injury prevention : journal of the International Society for Child and Adolescent Injury Prevention 1999 Sep;5(3):214-216.

(23) Macarthur C, Dougherty G, Pless IB. Reliability and validity of proxy respondent information about childhood injury: an assessment of a Canadian surveillance system. American Journal of Epidemiology 1997 May 1;145(9):834-841.

(24) Macarthur C, Dougherty G, Pless IB. Reliability and validity of proxy respondent information about childhood injury: an assessment of a Canadian surveillance system. Am J Epidemiol 1997;145(9):834-41.

(25) Hayes RJ, Bennett S. Simple sample size calculation for cluster-randomized trials. International journal of epidemiology 1999 Apr;28(2):319-326.

(26) Macarthur C, Hu X, Wesson DE, Parkin PC. Risk factors for severe injuries associated with falls from playground equipment. Accid Anal Prev 2000;32(3):377-82.

(27) Thompson S, Pike S, Hardy R. The design and analysis of paired cluster randomized trials: an application of meta-analysis techniques. Statistics in medicine 1997;16:2063-2079.

(28) Raudenbush S. Random effects model. In: Cooper H, Hedges L, editors. The Handbook of Reseach SynthesisNew York: Russell Sage Foundation; 1994.
