# Supplementary material for: School Playground Surfacing and Arm Fractures in Children: A Cluster Randomized Trial Comparing Sand to Wood Chip Surfaces
Source: PLoS Med. 2009 Dec 15;6(12):e1000195. doi: 10.1371/journal.pmed.1000195 (PMC2784292; doi:10.1371/journal.pmed.1000195)
Supplement: Text S2 — CONSORT statement checklist. (0.07 MB PDF) [file pmed.1000195.s002.pdf]

[Print] [Close]

School playground surfacing - a randomised prospective comparison of injury rates on granite sand versus wood chip surfaces

**ISRCTN**	ISRCTN02647424
**ClinicalTrials.gov identifier**	NCT00213174
**Public title**	School playground surfacing - a randomised prospective comparison of injury rates on granite sand versus wood chip surfaces
**Scientific title**	
**Acronym**	N/A
**Serial number at source**	MCT-70318
**Study hypothesis**	Injury rates (arm fracture, head injury and other injuries) among school children are equal on granitic sand and wood fibre (fibar) playground surfaces.
**Ethics approval**	Ethics approval received from the Hospital for Sick Children - University of Toronto - Research Ethics Board, on the 14th May 2004.
**Study design**	Randomised controlled trial
**Countries of recruitment**	Canada
**Disease/condition/study domain**	Children's fractures from falling off play equipment.
**Participants - inclusion criteria**	1. School children<br>2. Ages 5 - 11, either sex<br>3. Injured on falls from play equipment during supervised school hours
**Participants - exclusion criteria**	Children injured when school is not in session.
**Anticipated start date**	01/01/2005
**Anticipated end date**	31/12/2006
**Status of trial**	Completed
**Patient information material**	
**Target number of participants**	34 schools
**Interventions**	Granite sand playground surfaces versus wood fibre engineered playground surfaces.
**Primary outcome measure(s)**	Fracture rates, upper extremity
**Secondary outcome measure(s)**	Head injury, all injury
**Sources of funding**	Canadian Institutes of Health Research (CIHR) (Canada) - http://www.cihr-irsc.gc.ca (ref: MCT-70318)
**Trial website**	
**Publications**	
**Contact name**	**Dr Andrew William Howard**
Address	Hospital for Sick Children

	Orthopaedic Surgery and Population Health<br>555, University Avenue<br>Room S-107
City/town	Toronto, Ontario
Zip/Postcode	M5G 1X8
Country	Canada
Tel	+1 416 813 6439
Fax	+1 416 813 6414
Email	andrew.howard@sickkids.ca

**Sponsor**	Hospital for Sick Children, Toronto (Canada)
Address	555 University Avenue
City/town	Toronto, Ontario
Zip/Postcode	M5G 1X8
Country	Canada
Tel	+1 416 813 1500
Fax	+1 416 813 5393
Email	andrew.howard@sickkids.ca

**Date applied**	10/08/2005
**Last edited**	11/12/2007
**Date ISRCTN assigned**	10/08/2005
